# Comparative analysis of standard and contrast elastic resistance band training effects on physical fitness in female adolescent handball players

**DOI:** 10.5114/biolsport.2024.134143

**Published:** 2024-01-02

**Authors:** Mehrez Hammami, Piotr Zmijewski

**Affiliations:** 1 Research Laboratory (LR23JS01) «Sport Performance, Health & Society», Higher Institute of Sport and Physical Education of Ksar Saîd, University of Manouba, Tunis, Tunisia; 2 Higher Institute of Sport and Physical Education of Ksar Said, University of “La Manouba”, Tunis, Tunisia; 3 Jozef Pilsudski University of Physical Education in Warsaw, 00-809 Warsaw, Poland

**Keywords:** Sprint, Change of direction, Jump, Force-velocity test, Upper and lower limb strength, Repeated sprint ability

## Abstract

This study aimed to compare the effects of two elastic band 10-week training programmes on the athletic performance in adolescent female handball players. Participants aged 16.0 ± 0.5 years were randomly assigned to control (CNT, n = 12), standard elastic band (SEB, n = 12), or contrast elastic band (CEB, n = 12) programmes, each performed twice a week supplementing the regular training. The sprint (10 m and 20 m), modified Illinois change-of-direction test (COD), squat jump (SJ), countermovement jump (CMJ), standing long jump (SLJ), back extensor strength (BES), medicine ball throw (MBT), 1-RM bench press, 1-RM half squat, repeated sprint ability, and force-velocity (F-V) tests were measured before and after the intervention. Both CEB and SEB similarly improved sprint (p < 0.01 and p < 0.01) and COD (p < 0.001 and p < 0.01) when compared to CNT. Jumping performance improved significantly (SJ p < 0.01; CMJ p < 0.05) only in CEB, compared to CNT. Strength improved in both experimental groups (p < 0.01; ES: 0.73 < d < 1.59) compared to CNT, and there was a greater increase for CEB than SEB (p < 0.05) in the medicine ball throw (Table 3). Both CEB and SEB increased all RSA scores compared to CNT (p < 0.01; ES: 0.10 < d < 1.22), without significant difference between them. All F-V scores increased significantly in CEB and SEB compared to CNT (p < 0.01; ES: 0.45 < d < 2.47). In addition, CEB showed substantial gains in performance for PP_abs_, PP_rel_, and F0 (p < 0.001, p < 0.001 and p < 0.05, respectively) compared to SEB. Ten-week elastic band training conducted within the competitive season improved limb strength, power and F-V profile in female handball players, with a superior effect of the contrast elastic band training mode for upper-limb strength and F-V characteristics.

## INTRODUCTION

Handball, a physically demanding team sport, necessitates a combination of strength, power, speed, agility and others [1–5]. To excel in this sport, athletes must possess a well-rounded physical profile, including optimal muscular strength and power [6]. Consequently, training methods that enhance these attributes are pivotal for the development of adolescent female handball players [7–9].

One such increasingly popular training method is elastic band training, offering versatility and accessibility for resistance training. It provides various benefits, including accommodating resistance, enhancing joint stability, and increasing muscle activation [8–12]. Recently, researchers and practitioners have explored diverse elastic band training variations to maximize its effectiveness and tailor it to specific athletic needs [8–12].

A notable variant is contrast elastic band training, which combines standard elastic bands with those of higher resistance or different elastic properties [9]. This method capitalizes on the post-activation potentiation (PAP) phenomenon, where a high-intensity contraction is followed by subsequent performance improvement in explosive activities [13]. By integrating contrast elastic bands into training protocols, athletes may achieve greater muscular power and improved performance compared to traditional elastic band training alone [9].

Despite extensive studies on elastic band training in various athletic populations [8, 9, 14, 15], limited research has focused specifically on its effects on adolescent female handball players. Understanding the potential benefits and distinctions between standard elastic band training and contrast elastic band training in female handball players is essential for development of an evidence-based training programme that optimizes their athletic performance.

Hence, this study’s objective was to compare two types of elastic band strength training methods aimed at developing muscle force, explosiveness, muscle power, and assessing sprint, change of direction, vertical and horizontal jump, strength, power, and RSA scores. This comparison is critical for designing an evidence-based training programme tailored to a crucial phase in the playing season.

## MATERIALS AND METHODS

### Participants

A total of forty-five female players were selected from three national handball teams, all affiliated with a sports school. Their demographic information is as follows: age (16.2 [0.3] years), body mass (63.8 [5.3] kg), height (1.66 [0.3] m), and body fat percentage (23.4% [2.1%]). On average, these athletes possessed six years of experience in handball competition.

Approval for all procedures involving human subjects was obtained from the University Institutional Review Committee, ensuring ethical compliance. Informed consent, encompassing both verbal and written explanations of the experimental protocol, its potential risks, and benefits, was acquired from the athletes and their legal guardians. Participants were assured that they could withdraw from the trial at any point without incurring any penalties.

Prior to their participation, all athletes underwent a comprehensive examination by the team physician. This examination focused on identifying any conditions that might preclude them from engaging in plyometric training, particularly recent muscular or joint injuries. Importantly, all participants were found to be in good health.

### Study Design

In the week preceding the intervention, players underwent two familiarization sessions lasting 80 to 90 minutes each, aimed at acquainting them with all test procedures. The actual measurements were conducted over four days: both immediately before and four days after the final plyometric training session.

To ensure consistency and accuracy, several protocols were followed. Participants refrained from engaging in strenuous exercise for a 24-hour period prior to testing, and abstained from consuming any food or caffeine-containing beverages for at least two hours before testing. Additionally, a standardized warm-up was implemented before all tests, comprising 10 to 20 minutes of low-to moderate-intensity aerobic exercise and dynamic stretching.

The testing sequence was as follows: On the first test day, anthropometric measurements were taken, followed by a 30-m sprint and the modified Illinois change-of-direction test. Each of these tests allowed for three trials, with a 6- to 8-minute recovery period between trials, and the best performance times were recorded using paired photocells (Microgate, Bolzano, Italy). The second day was dedicated to jumping assessments, including the squat jump and countermovement jump, followed by 1-RM bench press and 1-RM half squat measurements. On the third day, back extensor strength assessments were conducted, followed by the standing long jump and a force-velocity test. The fourth day encompassed the medicine ball throw and the evaluation of repeated sprint ability.

### Anthropometry

Anthropometric measurements included standing and sitting body height (stadiometer accuracy of 0.1 cm; Holtain, Crosswell, Crymych, Pembrokeshire, United Kingdom) and body mass (0.1 kg; Tanita BF683W scales, Munich, Germany). The overall percentage of body fat was estimated from the biceps, triceps, subscapular, and suprailiac skinfolds, using the Durnin and Womersley [16] for children and adolescent females:

% Body fat = (495/D)-450

where D = 1.1369–0.0598 (log sum of 4 skinfolds)

Maturity offset status was calculated from peak height velocity [17]: Maturity offset = −9.38 + (0.000188 × leg length × sitting height) + (0.0022 × age × leg length) + (0.00584 × age × sitting height) + (0.0769 × weight / height ratio)

### 20 m sprint performance

During the sprint tests, players initiated the sprint from a split stance standing position, with their front foot positioned 0.2 m from the first photocell beam. Upon receiving the command, they sprinted for a distance of 20 m. Split times were recorded at both the 10-m and 20-m marks for subsequent analysis [18]. Notably, the reliability of these measurements was high, with intra-class correlation coefficients (ICC) of 0.91 for the 10-m sprint and 0.90 for the 20-m sprint. The coefficients of variation (CV) for these measurements were also low, standing at 2.6% for the 10-m sprint and 2.3% for the 20-m sprint.

### Change of direction performance

To measure change of direction performance, the modified Illinois test was used [19]. It utilized a configuration of four cones to delineate the change-of-direction area. In this test, players were instructed to sprint 5 m on command, make a turn, return to the starting line, and then navigate through the four markers with swerving movements to complete two additional 5-m sprints. While no specific technique was prescribed, players were directed to complete the test as swiftly as possible while avoiding crossing over the markers. If an athlete inadvertently “cut” a marker during the task, the trial was repeated. Three trials were permitted for both the 30-m sprint performance and the modified Illinois test, with a recovery period of 6 to 8 minutes between trials. The best time performances were recorded using paired photocells (Microgate, Bolzano, Italy). The results demonstrated strong reliability, with an intra-class correlation coefficient (ICC) of 0.91, indicating consistent measurements. The coefficient of variation (CV) was low, at 2.2%, attesting to the precision and reliability of the test.

### Vertical jumping

To measure jump height, the Optojump System (Microgate SARL in Bolzano, Italy), was utilized. Under the supervision of researchers, participants performed two types of vertical jumps: the squat jump (SJ) and the countermovement jump (CMJ). For the squat jump, participants initiated the jump with their knees bent at a 90-degree angle before executing the jump. For the countermovement jump, individuals commenced from a standing position, swiftly bending their knees to a 90-degree angle, and then launched themselves into the jump.

The reliability of these jump height measurements was noteworthy, with intra-class correlation coefficients (ICCs) of 0.88 for the squat jump (SJ) and 0.89 for the countermovement jump (CMJ). Furthermore, the coefficients of variation (CVs) for these measurements were 8.7% for the squat jump and 7.9% for the countermovement jump.

### Standing long jumping

In the beginning posture, players stood with their feet at the same height, legs bent and arms back, horizontal. Participants countermoved with their arms and legs as soon as the command ready, set, go was given before leaping as high as they could in the air. Participants could not fall forward or backward, and they had to land with both feet at the same time. With a tape measure, we measured the horizontal distance to the closest millimetre between the starting line and the heel of the back foot. The ICC is 0.88. The CV value is 8.5%.

### Strength performance

Determining an individual’s one-repetition maximum (1-RM) for both the half squat and bench press involved a methodical practice regimen. Participants executed a series of lifting sets, with up to six sets in total, and observed a four-minute recovery period between sets. During this process, the weight loads were systematically increased, typically by 5% to 10% of the participant’s 1-RM for the bench press and 10% to 20% of the 1-RM for the half squats. The objective was to progressively increase the weight until reaching the point at which the participant could perform just one successful repetition. This weight, defined as the 1-RM, represents the maximum load that an individual can lift for a single repetition [20, 21].

### The Force-Velocity Profile

A Sweden Monark, model 894 E was used for this test, which aims to determine the strength of the upper limbs from the most intense short sprint runs (maximum duration 7 s) of pedalling against a load of 1.5%, 2%, 2.5%, 3%, 4.5%, and 2.5% of body mass in the upper limb. Peak power (W), peak power per kilogram of body mass (W/kg), maximum pedalling velocity (rpm), and maximum braking force are the terms used to describe performance (N). For more details, see Chelly et al. 2010 [22].

### Medicine ball throw

The test was performed using 21.5-cm diameter, 3-kg rubber medicine balls (Tigar, Pirot, Serbia) powdered with magnesium carbonate. A familiarization session included a brief description of the optimal technique [23]. The seated player grasped the medicine ball with both hands, and on a signal forcefully pushed the ball from the chest. The score was measured from the front of the sitting line to the powder-marked spot where the ball landed. The ICC is 0.86. The CV value is 16.7%.

### Repeated sprint ability (RSA) test

The repeated sprint ability consisted of recording six sets of two 20-m sprint round trips using paired photocells. Each sprint was performed once every 20 s [24]. Four scores were determined: best and total sprint times and fatigue index [24].

### Training programme

All players engaged in handball training five to six times per week and participated in one official game per week. Standard training sessions typically lasted 80 to 100 minutes, focusing on various skill activities, offensive and defensive strategies, and 25 to 30 minutes of continuous play, with minimal interruptions by the coach.

Both contrast elastic band training (CEB) and standard elastic band training (SEB) were integrated into their training regimens for ten weeks, replacing certain low-intensity technical-tactical handball drills on Tuesdays and Thursdays (as detailed in Table 2). The training programme was designed to be progressive and spanned a total of 20 sessions. During these biweekly sessions, which took place on Tuesdays and Thursdays, eight exercises were performed, with four targeting the upper limbs (flies, rows with high elbows, trunk rotation, and standing presses) and four focusing on the lower limbs (knee extension, knee flexion, half-squats, and hip adduction). The exercises alternated between upper and lower limb exercises for all experimental groups. Thera-Bands, an elastic band system comprising four latex bands (red, green, blue, and black), were used. The length and load of each elastic band are documented in Table 2. Both CEB and EB groups performed the same exercises, the same number of sets, and the same number of repetitions. The key distinction between CEB and EB was in the repetition scheme. EB completed 12 repetitions with the same load (i.e., 12 reps at 250% of the length of the elastic band), while CEB performed 6 repetitions at 250% of the length of the elastic band, followed by 6 repetitions at 100% of the length of the elastic band.

**TABLE 1:** Physical characteristics of intervention and control groups (mean ± SD).

	Age (years)	Body mass (kg)	Height (cm)	% Body fat	APHV (years)	Predicted years from APHV
CEB group (n = 12)	16.2 ± 0.3	64.3 ± 4.0	167.1 ± 3.7	23.3 ± 1.0	3.3 ± 0.4	12.9 ± 0.3
SEB group (n = 12)	16.2 ± 0.4	63.8 ± 3.3	165.5 ± 3.2	23.0± 1.1	3.3 ± 0.3	13.0 ± 0.4
CNT group (n = 12)	16.3 ± 0.3	63.5 ± 4.4	167.6 ± 3.6	23.9 ± 1.4	3.3 ± 0.4	13.0 ± 0.4

APHV: age of peak height velocity; CEB = contrast elastic band; SEB = standard elastic band; CNT = control

**TABLE 2:** Training programme of both contrast and standard elastic band training.

Exercises	Week 1	Week 2	Week 3	Week 4	Week 5
Load	Sets × Reps				

*Upper limb*					

Resistance at 250 and 100% elongation	Red elastic band (3.2 and 1.8 kg)	Green elastic band (4.4 and 2.3 kg)			Blue elastic band (6 and 3.2 kg)

Flies, row with high elbows, trunk rotation and standing press	3 × (6+6)	3 × (6+6) 4 × (6+6) 5 × (6+6)			3 × (6+6)

*Lower limb*					

Resistance at 250 and 100% elongation	Red elastic band folded (6.4 and 3.6 kg)	Green elastic band (8.8 kg and 4.6 kg)			Blue elastic band folded (12 and 6.4 kg)

Knee extension and flexion, half squat and hip adduction	3 × (6+6)	3 × (6+6) 4 × (6+6) 5 × (6+6)			3 × (6+6)

	**Week 6**	**Week 7**	**Week 8**	**Week 9**	**Week 10**

*Upper limb*					

Resistance at 250 and 100% elongation	Blue elastic band (6 and 3.2 kg)			Black elastic band (8 and 4.4 kg)	

Flies, row with high elbows, trunk rotation and standing press	4 × (6+6)	5 × (6+6)	3 × (6+6)	4 × (6+6)	5 × (6+6)

*Lower limb*					

Resistance at 250 and 100% elongation	Blue elastic band folded (12 and 6.4 kg)			Black elastic band folded (16 and 8.8 kg)	

Knee extension and flexion, half squat and hip adduction	4 × (6+6)	5 × (6+6)	3 × (6+6)	4 × (6+6)	5 × (6+6)

Participants performed the first 6 reps at 250% elongation of the initial length of the elastic band directly followed by 6 repetitions at 100% elongation.

Each training session commenced with a 15-minute warm-up and lasted for 30 minutes, resulting in a total session duration of 45 minutes.

### Statistical analyses

Statistical analyses were carried out using the IBM SPSS Statistics (version 20.0, IBM Corp., Armonk, NY, USA). Normality of all variables was verified using the Kolmogorov-Smirnov procedure. Levene’s test was used to determine homogeneity of variance. Means and standard deviations (SDs) were calculated using standard statistical methods. Training-related effects were assessed by 2-way analyses of variance with repeated measures (group × time). If a significant F value was observed, Tukey’s post hoc procedure was applied to locate pair-wise differences. A p ≤ 0.05 was accepted as the criterion of statistical significance, whether a positive or a negative difference was seen (i.e., a 2-tailed test was adopted). Effect sizes were reported for a main effect of group, a main effect of time and a main effect of group × time interaction; values were classified as small (0.00 ≤ d ≤ 0.49), medium (0.50 ≤ d ≤ 0.79), or large (d ≥ 0.80), as suggested by Cohen [25]. The reliabilities of all dependent variables were assessed by calculating 2-way mixed intra-class correlation coefficients (ICC), and coefficients of variation (CV).

## RESULTS

Intraclass correlation coefficients assessing the reliability and the coefficient of variance of sprint, COD, jump, and medicine ball throw tests are displayed in each protocol test. Test results before and after interventions are outlined in Tables 3 and 4. No significant differences were observed before training between groups. After intervention, both CEB and SEB significantly increased sprint (p < 0.01; d = 0.88) and COD (p < 0.001; d = 0.97) relative to C, without a difference between them. Jump performance improved significantly (SJ (p < 0.01); CMJ (p < 0.05)) only in CEB relative to C. All strength test results increased significantly in both experimental groups (0.73 < d < 1.59) relative to C, with a slight difference between CEB and SEB (p < 0.05) in medicine ball throw (Table 3). Both CEB and SEB significantly increased all RSA scores relative to the controls (0.10 < d < 1.22), without a difference between them (Table 4). All force-velocity scores increased significantly in CEB and SEB relative to C (0.45 < d < 2.47). In addition, the CEB showed substantial gains of performance for PP_abs_, PP_rel_, and F0 (p < 0.001, p < 0.001 and p < 0.05, respectively) relative to SEB (Table 4).

**TABLE 3:** Sprint, change of direction, jump, and upper limb strength performance in all groups before and after the 10-week intervention.

	CEB Group			SEB Group			CNT Group			ANOVA group * time interaction	

	Pre	Post	∆	Pre	Post	∆	Pre	Post	∆	P	d
**Sprint**											

10 m (s)	2.24 ± 0.05	2.10 ± 0.06 μμ	6.1 ± 1.6	2.24 ± 0.06	2.09 ± 0.05 αα	7 ± 1.1	2.24 ± 0.06	2.20 ± 0.08	1.7 ± 2.4	0.003	0.88

20 m (s)	3.69 ± 0.10	3.24 ± 0.10 μμμ	12.3 ± 1.1	3.76 ± 0.10	3.36 ± 0.09 ααα	10.5 ± 1.1	3.75 ± 0.05	3.64 ± 0.07	1.9 ± 1.2	0.003	0.88

**COD**											

Illinois-MT (s)	13.11 ± 0.37	11.28 ± 0.77 μμμ	6.3 ± 1.1	13.13 ± 0.23	12.40 ± 0.20 αα	5.6 ± 0.3	13.16 ± 0.39	13.05 ± 0.38	0.7 ± 0.2	0.001	0.97

**Jump**											

SJ (cm)	23.2 ± 2	27.3 ± 2.3 μμ	17.9 ± 4.1	22.4 ± 1.7	26.4 ± 1.4	17.3 ± 6.3	22.8 ± 2.4	23.7 ± 1.9	4.4 ± 4.8	0.012	0.75

CMJ (cm)	23.1 ± 2.5	28 ± 1.9 μ	19.4 ± 2.9	23.4 ± 1.6	27.5 ± 2.1	17.5 ± 2.7	24 ± 2.3	25.2 ± 2.1	4.4 ± 3.8	0.007	0.81

Standing long jump (m)	1.50 ± 0.10	1.60 ± 0.10	6.8 ± 4.6	1.50 ± 0.13	1.59 ± 0.11	6.5 ± 3.5	1.53 ± 0.16	1.71 ± 0.18	12.8 ± 14.1	0.430	0.32

**Strength**											

Back extensor (N)	737 ± 92	1149 ± 92 μμμ	56.5 ± 6.6	753 ± 58	1170 ± 143 ααα	55.4 ± 14.6	744 ± 35	866 ± 83	16.2 ± 7.2	0.000	1.59

Medicine ball throw (m)	3.33 ± 0.36	5.03 ± 0.58 μμμ β	62.4 ± 11.9	3.38 ± 0.37	4.35 ± 0.46 αα	28.7 ± 4.6	3.26 ± 0.57	3.49 ± 0.62	7.41 ± 0.59	0.000	1.23

1-RM bench press (kg)	41.2 ± 10.7	60.4 ± 9 μμ	54.8 ± 25.1	40.7 ± 39.5	54.8 ± 12 α	40.7 ± 39.5	40.2 ± 11.5	44.2 ± 11.2	5.8 ± 9.2	0.016	0.73

1-RM half squat (kg)	72.3 ± 9.1	96.8 ± 11.2 μμ	34.6 ± 12.8	78.8 ± 12	96 ± 8.9 ααα	23.1 ± 10.2	72.6 ± 16.6	82.2 ± 13.4	17.8 ± 17.3	0.012	0.75

COD = change of direction; CEB = contrast elastic band group; SEB = standard elastic band group; CNT = control group; T-Modified = modified change of direction T-test; CMJ = counter-movement jump; CMJA = counter-movement jump with arm swing; µ = significant difference between CEB and CNT at p < 0.05; µµ = significant difference between CEB and CNT at p < 0.01; µµµ = significant difference between CEB and CNT at p < 0.001; α = significant difference between SEB and CNT at p < 0.05; αα = significant difference between SEB and CNT at p < 0.01; ααα = significant difference between SEB and CNT at p < 0.001; β = significant difference between CEB and SEB at p < 0.05.

**TABLE 4:** Repeated sprint ability and force-velocity performance in all groups before and after the 10-week intervention.

	CEB Group			SEB Group			CNT Group			ANOVA group * time interaction	

	Pre	Post	∆	Pre	Post	∆	Pre	Post	∆	P	d
**RSA**											

RSA-BT (s)	7.55 ± 0.12	7.31 ± 0.12 µµµ	3.1 ± 0.8	7.55 ± 0.08	7.31 ± 0.09 ααα	3.1 ± 0.4	7.55 ± 0.08	7.51 ± 0.07	0.5 ± 0.4	0.000	1.02

RSA-MT (s)	7.67 ± 0.10	7.42 ± 0.11 µµµ	3.2 ± 0.6	7.67 ± 0.04	7.44 ± 0.07 ααα	3.1 ± 0.2	7.71 ± 0.07	7.67 ± 0.06	0.6 ± 0.5	0.000	1.22
RSA-TT (s)	46 ± 0.58	44.51 ± 0.64 µµµ	3.2 ± 0.6	46.04 ± 0.41	44.61 ± 0.42 ααα	3.1 ± 0.2	46.29 ± 0.43	46.03 ± 0.35	0.6 ± 0.5	0.000	1.22

RSA-FI	1.59 ± 0.40	1.46 ± 0.34 µµµ	5.4 ± 19.8	1.70 ± 0.37	1.67 ± 0.49 αα	1.7 ± 20.1	2.21 ± 0.64	2.18 ± 0.59	0.8 ± 20.5	0.920	0.10

**Force-velocity test**											

PPabs (W)	145.8 ± 18.2	271.8 ± 24.5 µµµ βββ	89.7 ± 35.1	144.7 ± 23.4	197.6 ± 21.8 ααα	37.8 ± 8.8	142.4 ± 23.8	162.3 ± 22.4	14.7 ± 8.3	0.000	2.47

PPrel (W · kg−1)	2.28 ± 0.32	4.23 ± 0.36 µµµ βββ	86.7 ± 35.1	2.27 ± 0.38	3.11 ± 0.37 αα	37.8 ± 8.8	2.23 ± 0.28	2.55 ± 0.23	14.7 ± 8.3	0.000	2.20

V0 (rpm)	90.4 ± 16.9	103.2 ± 9.5 µµ	17.4 ± 22.9	90.5 ± 16.6	102.4 ± 13.7 αα	15.6 ± 18.6	85.7 ± 17.7	84.8 ± 9	2.6 ± 22	0.190	0.45

F0 (N)	6.7 ± 1	10.2 ± 1.3 µµµ β	55.5 ± 22.3	6.5 ± 0.7	8.9 ± 1.5 αα	37.3 ± 19.7	6.4 ± 1.2	7.1 ± 0.7	13.2 ± 25.6	0.000	1.13

CEB = contrast elastic band group; SEB = standard elastic band group; CNT = control group; RSA = repeated sprint ability; BT = best time; MT = mean time; TT = total time; FI = fatigue index; PP = peak power; V0 = maximal pedalling velocity; F0 = maximal breaking force; µµ = significant difference between CEB and CNT at p < 0.01; µµµ = significant difference between CEB and CNT at p < 0.001; αα = significant difference between SEB and CNT at p < 0.01; ααα = significant difference between SEB and CNT at p < 0.001; β = significant difference between CEB and SEB at p < 0.05; βββ = significant difference between CEB and SEB at p < 0.001.

## DISCUSSION

In our study, we investigated the effects of two types of strength training, namely contrast elastic band training (CEB) and standard elastic band training (SEB), on various performance parameters critical for adolescent female handball players during a crucial phase in the playing season. Most measures demonstrated comparable gains in both groups, except for the medicine ball throw and upper limb force-velocity scores.

In line with findings from other researchers, our study highlights the advantages of applying an elastic band programme in both standard and contrast training for sprint performance. Our results align with those of Hammami et al. [9], who observed significant improvements in 5 m (p = 0.02, d = 0.80 (large)) and 30 m (p = 0.02, d = 2.60 (large)) in young female athletes. Also, the authors found increases in all sprint performances after 10-week elastic band training in young female handball players [15]. Similarly, when examining U-17 male soccer players, it was reported that both 8-week contrast and standard strength training programmes improved sprint performance (specifically in the 5-m and 40-m sprints), with no significant differences between the two groups [26]. The improvement in sprint performance observed in female adolescent handball players following both CEB and SEB can be attributed to the improved neuromuscular adaptations. Elastic band training necessitates the activation of stabilizer muscles and fosters neuromuscular coordination. These bands offer variable resistance, thereby challenging the muscles to adapt and coordinate their contractions effectively. This, in turn, could result in improved recruitment of motor units, synchronization, and overall enhancement of neuromuscular efficiency, all of which are crucial for sprint performance.

Handball, known for its fast-paced nature, demands quick changes in movement direction [1, 2]. Female handball players must navigate the court, evade opponents, and position themselves for both offensive and defensive actions. Effective change of direction is essential for swift reactions to in-game situations, making sharp cuts, and maintaining control over movements [1, 2]. Elastic band training has been recommended to enhance fundamental handball movements, including sprints and COD actions [9, 15]. Our findings support the positive impact of elastic band training on COD abilities in handball players, with significant improvements observed in both CEB and SEB groups (p < 0.001 and p < 0.01 respectively). These results are consistent with those of Aloui et al. [14], who reported similar improvements in junior male handball players after an 8-week programme of strength training with elastic bands. However, our findings contradict those of Anderson et al. [27], who observed no significant change in agility after a 6-week elastic band strength training programme in young female handball players. These discrepancies may be attributed to variations in test methodologies, intervention durations, and the specific characteristics of the training protocols used in the studies.

The discrepancies in the results may be attributed to variations in test techniques and interventions, such as the frequency, duration, and progression of training relative to the playing season. The observed improvements in change of direction (COD) performance among female adolescent handball players can be attributed to a combination of factors, including increased strength and power, improved proprioception and balance, enhanced neuromuscular coordination, greater agility and quickness, improved eccentric strength, and the transfer of these gains to sport-specific movements through elastic band training. Both standard and contrast formats of elastic band training contribute to the enhancement of the physical attributes and motor skills necessary for executing effective and efficient changes of direction on the handball court.

Concerning jump performance, we observed that the CEB group achieved small to large improvements across the measured jump performance parameters (with effect sizes ranging from d = 0.31 to d = 0.82) compared to the control group (p < 0.05; p < 0.01 in CMJ and SJ respectively). These findings align with those of Hammami et al. [9], who observed significant increases in jump performance (SJ, CMJ, and CMJ with arm swing) following contrast elastic band training in female handball players. Our results did not reveal a significant difference between SEB and the control group in jump performance. This contradicts the findings of another study [15], which reported medium-sized increases in SJ and CMJ performance after elastic band training in female handball players. These discrepancies between studies may arise from various factors, including differences in the type of elastic band used (type, length, colour), as well as variations in the demographic characteristics (age and sex) of the participants. It seems that both contrast and standard elastic band training methods can effectively enhance jump abilities, with the contrast format capitalizing on the potentiation effect and power development aspects of the training stimulus, resulting in slightly superior jump performance improvements in female adolescent handball players.

Strength and power are pivotal in enhancing handball performance. The sport demands explosive actions such as throwing, jumping, sprinting, and tackling [2–4, 28]. Developing strength and power empowers players to generate greater force and speed, resulting in more forceful throws, higher jumps, and swifter movements on the court [2–4, 28]. In this context, both training programmes, CEB and SEB, showed notable improvements in various performance traits.

Firstly, after the band training there were substantial enhancements in back extensor strength, a key component in athletic movements, with an effect size of 1.59. Additionally, players demonstrated significant improvement in the medicine ball throw, which measures upper body explosive strength and power, with an effect size of 1.23. Moreover, the evaluation of maximal strength through the 1-RM bench press and 1-RM half squat also showed considerable improvement, with effect sizes of 0.73 and 0.75, respectively.

When comparing CEB and SEB, it was noted that CEB displayed a slight, but significant advantage in the medicine ball throw. This suggests that contrast elastic band training had a more pronounced impact on medicine ball throw performance than standard elastic band training. Several research studies have consistently highlighted improvements in both upper and lower limb strength, regardless of variations in the specific tests and evaluation instruments used. For instance, Anderson et al. [27] reported enhanced ball throwing velocity and bench press performance following a 6-week elastic band strength training programme. Similarly, Mascarin et al. [29] observed an increase in the average power of shoulder internal rotation, along with improved ball throwing speed from a standing position and during jumping, after a 6-week elastic band strength training programme in young female handball players. In another study by Mascarin et al. [12], elastic band strength training contributed to enhanced muscular strength in the external rotator muscles, promoting muscular balance in female youth handball players. The improvements in back extensor strength, medicine ball throw performance, 1-RM bench press, and 1-RM half squat observed after both CEB training and SEB training in female adolescent handball players can be attributed to various factors. These include the combination of increased muscle strength, improved power output, enhanced muscle activation and coordination, improved rate of force development, specificity of training, and increased joint stability and core strength. Collectively, these factors contribute to the observed enhancements in these crucial physical attributes. The selected studies suggest that a programme involving medicine ball workouts facilitates improvements in key components of physical performance [30]. The integration of varied means into the training process of pre-adolescent female athletes further optimises the impact of exercise influences. An intriguing avenue for exploration may involve a comparative analysis of the effects of these different training modalities.

In the context of handball, where short, maximum sprints interspersed with rapid recovery intervals are common during matches, the ability to repeatedly execute sprints is a critical fitness component. In this study, both the CEB and SEB groups demonstrated enhanced RSA scores compared to the C group, and there was no significant difference between CEB and SEB groups in this regard (Table 5). Several studies have reported an increase in RSA scores after training with elastic bands. This improvement in repeated sprint ability can be attributed to a combination of factors, including enhanced anaerobic capacity, increased muscular endurance, improved speed and acceleration, neuromuscular adaptations, enhanced recovery capacity, and mental toughness. These factors collectively contribute to the observed enhancements in the ability to perform repeated sprints, which is crucial in handball [24, 31–35]. The findings of this study also demonstrated substantial improvements in upper limb force-velocity performance in both the CEB and SEB groups compared to the C group. Effect sizes (d values) ranged from 0.45 to 2.47 across all scores, indicating a significant effect. Notably, there was a significant difference between CEB and SEB in favour of the former (Table 5). Similar results were obtained in the study by Gaamouri et al. in 2023 [8], where they observed increases in all upper limb force-velocity scores with effect sizes [d values) ranging from 0.56 to 1.66 after a 10-week strength training programme using elastic bands in youth handball players. Similarly, Aloui et al. in 2019 [10] reported an increase in upper limb force-velocity scores during an 8-week, in-season training programme using upper limb elastic bands in junior handball players, with effect sizes (eta squared) ranging from 0.183 to 0.591. The greater improvement in upper limb force-velocity test performance following CEB compared to SEB in female adolescent handball players can be attributed to the specific training stimulus provided by the contrast format. This effect occurs when the muscles experience a contrasting stimulus, such as going from a resisted movement to an unresisted movement [11, 36]. The bands provide resistance during the eccentric phase of the movement and then release tension during the concentric phase, resulting in a rapid transition to an unloaded movement [11, 36]. This contrast of resistance levels enhances the muscle’s ability to produce force and power, leading to greater improvements in upper limb forcevelocity test performance.

## CONCLUSIONS

In terms of practical implications, the study demonstrates that both the CEB and SEB groups exhibited significant improvements in various aspects of athletic performance over the 10-week intervention period and the findings provide insights into how coaches and athletes can apply the results of our study in designing effective training regimens. Nevertheless, it is noteworthy that specific measures of athletic performance, such as the medicine ball throw and forcevelocity performance, exhibited more substantial enhancements following the 10-week CEB regimen compared to the SEB training. These findings underline the potential advantages of contrast elastic band training (CEB) in targeting and enhancing particular athletic performance parameters in adolescent female handball players.

## Conflict of interest declaration

The authors declare no conflict of interest.
